# Synovial cell death is regulated by TNF-*α*-induced expression of B-cell activating factor through an ERK-dependent increase in hypoxia-inducible factor-1*α*

**DOI:** 10.1038/cddis.2017.26

**Published:** 2017-04-06

**Authors:** Jae-Wook Lee, Jiyoung Lee, Sung Hee Um, Eun-Yi Moon

**Affiliations:** 1 Department of Bioscience and Biotechnology, Sejong University, Seoul 05006, Korea; 2 Department of Molecular Cell Biology, Samsung Biomedical Research Institute, Sungkyunkwan University School of Medicine, Suwon, Kyunggi-do 16419, Korea

## Abstract

B-cell activating factor (BAFF) has a role in the maturation and maintenance of B cells and is associated with rheumatoid arthritis (RA). Here, we investigated whether tumor necrosis factor (TNF)-*α*-induced BAFF expression controls the survival of fibroblast-like synoviocytes (FLS) and whether their survival can be regulated by TNF-*α*-mediated upregulation of hypoxia-inducible factor (HIF)-1*α* using MH7A synovial cells transfected with the SV40 T antigen. More TNF-*α*-treated cells died compared with the control. Survival was increased by incubation with Z-VAD but inhibited after transfection with BAFF-siRNA. Both BAFF and HIF-1*α* expression were enhanced when MH7A cells were treated with TNF-*α*. TNF-*α*-induced BAFF expression decreased in response to HIF-1*α*-siRNA, whereas it increased under hypoxia or by overexpressing HIF-1*α*. The HIF-1*α* binding site on the BAFF promoter (−693 to −688 bp) was confirmed by chromatin immunoprecipitation assay to detect the −750 to −501 bp and −800 to −601 bp regions. The BAFF promoter increased in response to TNF-*α* treatment or overexpression of HIF-1*α*. However, TNF-*α*-induced BAFF expression and promoter activity decreased after treatment with the ERK inhibitor PD98059. Cell death was enhanced by PD98059 but was inhibited by overexpression of HIF-1*α*. Taken together, our results demonstrate that BAFF expression to control synovial cell survival was regulated by HIF-1*α* binding to the BAFF promoter, and suggest for the first time that HIF-1*α* might be involved in the production of inflammatory cytokines to regulate the physiological function of rheumatic FLS.

Synovial hyperplasia and destruction of cartilage and bone are characteristics of rheumatoid arthritis (RA).^1^ The synovial membrane is thin in a normal joint and consists of only a few cells. However, many cell types, including immune cells and synoviocytes, occur in a rheumatoid synovial membrane.^2^ Recruitment and accumulation of immune cells in joint tissue induces inflammation^3^ and the abnormal increase in the number of synoviocytes causes low oxygen tension.^4, 5^ Both inflammation and hypoxia are major microenvironmental features of RA.

Hypoxia-inducible factor-1*α* (HIF-1*α*), which is a regulator of angiogenesis, tumor growth, and glucose metabolism, is a well-known transcription factor in cancers.^6, 7^ HIF-1*α* also has an important role in the pathogenesis of RA.^8^ High expression levels of HIF-1*α* are detected in the intimal synovium of patients with RA and are localized in the nucleus and cytoplasm of synoviocytes.^9^ HIF-1*α* is normally degraded under normoxic conditions by the ubiquitin–proteasome pathway;^10^ however, it accumulates under normoxic conditions in an inflammatory environment.^11^ Various immune cells, including macrophages, T cells, B cells, and plasma cells are recruited to the layer that lines the synovium during the progression of RA.^12^ Although angiogenesis occurs, a malfunctioning vascular system maintains the hypoxic conditions.^13, 14^ Hypoxia-exposed macrophages produce additional quantities of proinflammatory cytokines, such as tumor necrosis factor (TNF)-*α*.^15^

RA is an immune disorder associated with many cytokines. Synovial macrophages and synoviocytes produce abundant pro- and anti-inflammatory cytokines, such as TNF-*α*, interleukin (IL)-1, IL-6, and transforming growth factor-*β*.^3, 16, 17^ TNF-*α* regulates other cytokines, destroys joint tissue,^18, 19^ and stabilizes HIF-1*α* under normoxic conditions.^20^

Fibroblast-like synoviocytes (FLS), which are components of the synovial membrane, have a crucial role in initiating RA. RA-FLS develop cancer cell-like characteristics, such as anchorage-independent growth, loss of contact inhibition, and an invasive phenotype.^21^ They also produce and release proinflammatory cytokines, matrix metalloproteinases, and growth factors that affect other cells.^22^ TNF-*α*-stimulated FLS express B-cell activating factor (BAFF), originally known as a B-cell proliferation and survival factor.^23^ BAFF is produced by immune cells (monocytes, dendritic cells, and macrophages),^24^ and also by non-immune cells, such as salivary gland epithelial cells,^25^ prostate epithelium,^26^ and FLS.^27^ Excess levels of BAFF are detected in patients with an autoimmune disease, and particularly in the synovial tissue of patients with RA.^28^ Increased B-cell survival in response to BAFF can be detrimental to patients with an autoimmune disorder. An excess B-cell response increases circulating autoantibody levels in patients with certain autoimmune disorders, such as systemic lupus erythematosus, Sjögren's syndrome, and RA.^27, 29, 30^ We reported previously that TNF-*α*-induced BAFF controls RA angiogenesis by regulating vascular endothelial growth factor (VEGF) expression in synoviocytes.^31^

Although HIF-1*α* and BAFF are highly expressed in the joints of patients with RA, the relationship between these two factors is not understood. In this study, we investigated whether TNF-*α*-induced BAFF expression controls synovial cell survival, and whether TNF-*α* regulates HIF-1*α* and BAFF expression through the extracellular-regulated kinase (ERK) pathway in TNF-*α*-stimulated MH7A synovial cells. We also describe the mechanism of action underlying BAFF expression, which is regulated in a HIF-1*α*-dependent manner in TNF-*α*-stimulated FLS.

## Results

### hBAFF expression is associated with the survival of synovial cells

**Figure 1:**
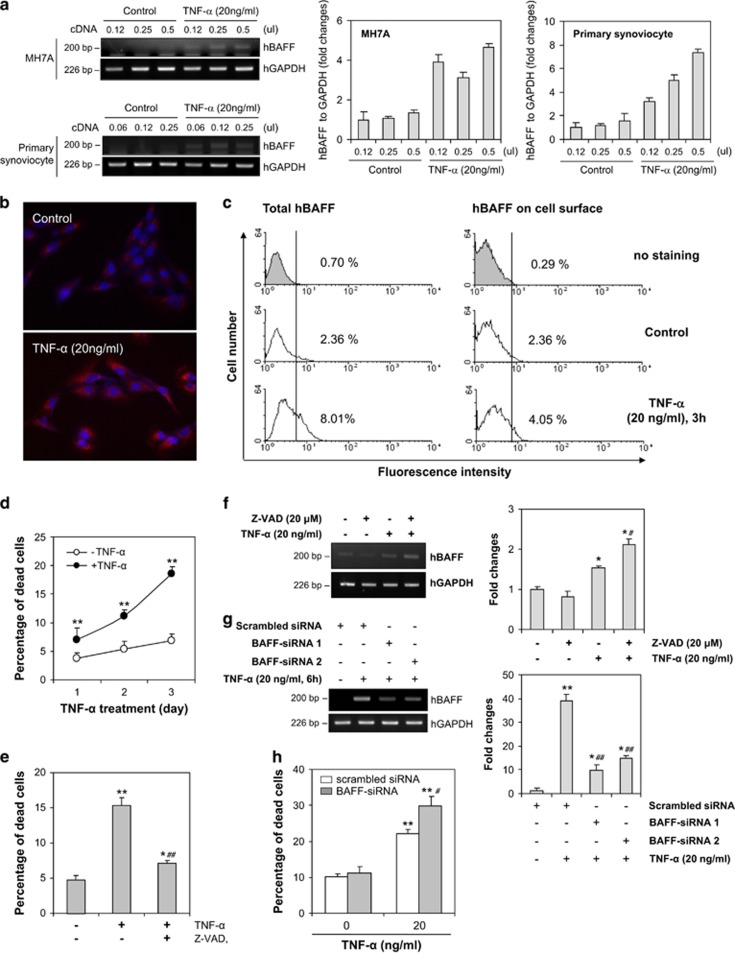
TNF-*α*-induced BAFF expression was associated with synovial cell survival. (**a**) MH7A cells (left top and middle) and fibroblast-like synoviocytes (FLS) from RA patient (left bottom and right) were stimulated with 20 ng/ml TNF-*α* for 6 h. RNA was isolated with TRIzol^TM^. hBAFF transcripts were measured by RT-PCR. Each band was quantified by using ImageJ 1.34 (**a**, middle and right). (**b**–**d**) MH7A cells were stimulated with 20 ng/ml TNF-*α*. Then, the cells were fixed or permeabilized and incubated with biotinylated rabbit anti-BAFF antibodies. BAFF expression was visualized by the incubation with phycoerythrin (PE)-conjugated streptavidin (**b** and **c**). The cells were observed under fluorescence microscope with × 400 magnification (**b**) or analyzed with flow cytometer (**c**). Dead cells were estimated with trypan blue exclusion assay (**d**). (**e** and **f**) MH7A cells were treated with TNF-*α* for 3 days (**e**) or 6 h (**f**) in the presence or absence of Z-VAD. Dead cells were estimated with trypan blue exclusion assay (**e**). hBAFF transcripts were measured by RT-PCR (**f**, left). Each band was quantified by using ImageJ 1.34 (**g**, right). (**g** and **h**) MH7A cells were transfected with hBAFF-siRNA and treated with TNF-*α*. Then, hBAFF transcripts were measured by RT-PCR (**g**, left). Each band was quantified by using ImageJ 1.34 (**g**, right). Dead cells were estimated with trypan blue exclusion assay (**h**). Data were the representative of four experiments. Data in the bar or line graph represent the means±S.E.M. *P<0.05; **P<0.01; significant difference as compared with TNF-*α*-untreated control. ^#^*P*<0.05; ^##^*P*<0.01; significant difference as compared with TNF-*α*-treated and Z-VAD-untreated control (**e** and **f** right) or TNF-*α*-treated and scrambled siRNA-treated control (**g** and **h** right)

FLS have a crucial role in initiating RA, and RA-FLS develop cancer cell-like characteristics;^21^ thus, we examined hBAFF expression and the effect of hBAFF on survival of synovial cells using MH7A synovial cells. Synovial cells isolated from patients with RA were treated with TNF-*α* for 1, 3, 6, 9, 12 h, and hBAFF expression was highest after the 6 h treatment (data not shown). We also confirmed that hBAFF expression was increased by stimulating FLS from patients with RA or MH7A synovial cells with TNF-*α* for 6 h (Figure 1a). TNF-*α*-induced BAFF was also observed by immunostaining and fluorescence-activated cell sorting analysis. hBAFF proteins were detected in the intracellular compartment and on the synovial cell surface (Figures 1b and c). Then, we studied the role of hBAFF expression in synovial cells. As synovial hyperplasia is a characteristic of RA, we examined cell death in TNF-*α*-treated MH7A cells using the trypan blue exclusion assay. The percentage of dead cells increased significantly after treatment with TNF-*α* (Figure 1d). In contrast, the percentage of dead cells decreased significantly after incubating the cells with TNF-*α* in the presence of Z-VAD (Figure 1e). hBAFF expression was enhanced by incubating the cells with TNF-*α* in the presence of Z-VAD (Figure 1f). We confirmed a role for hBAFF in the survival of synovial cells by inhibiting BAFF expression using BAFF-siRNA (Figure 1g). The percentage of dead cells increased significantly after transfection with hBAFF-siRNA (Figure 1h). These data demonstrate that hBAFF expression could be associated with the survival of synovial cells.

### hBAFF expression increases following TNF-*α* treatment of RA-FLS, MH7A cells

**Figure 2:**
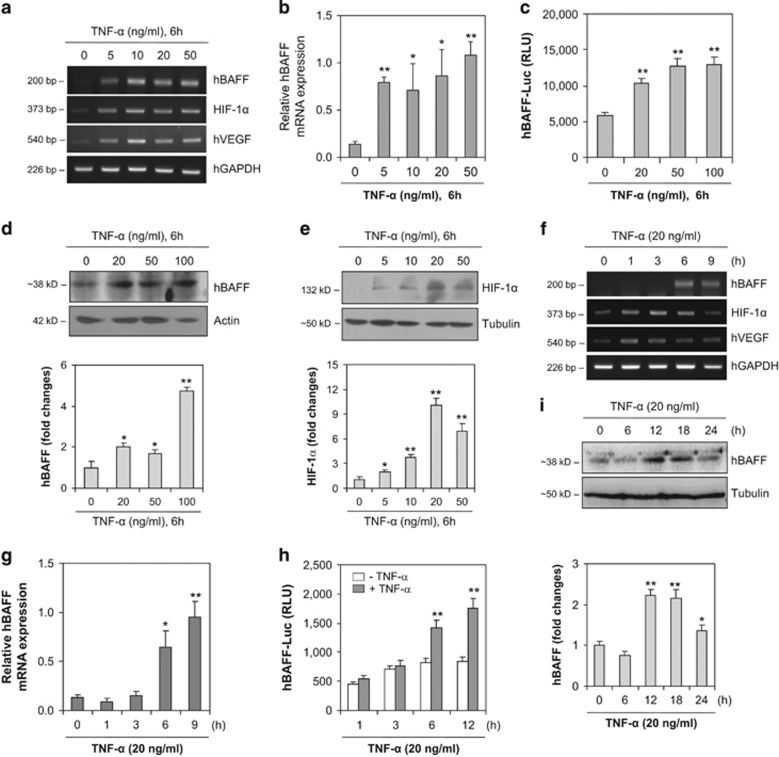
hBAFF expression was increased by the treatment with TNF-*α*, dose- and time-dependently. (**a**) and (**b**) MH7A cells were stimulated with various concentrations of TNF-*α* for 6 h. RNA was isolated with TRIzol^TM^. hBAFF transcripts were measured by RT-PCR (**a**) and real-time qPCR normalized to GAPDH expression (**b**). (**c**) MH7A cells were transfected with pGL3-hBAFF-Luc plasmid and stimulated with various concentrations of TNF-*α*. Luciferase activity of hBAFF promoter was measured by using luminometer. (**d** and **e**) MH7A cells were stimulated with various concentrations of TNF-*α* for 6 h. Cell lysates were prepared and western blotting was used to detect hBAFF (**d**, top) or HIF-1*α* (**e**, top). Each protein band was quantified by using ImageJ 1.34. (**f** and **g**) MH7A cells were stimulated with TNF-*α* for appropriate time. RNA was isolated with TRIzol^TM^. hBAFF transcripts were measured by RT-PCR (**f**) and real-time qPCR normalized to GAPDH expression (**g**). (**h**) MH7A cells were transfected with pGL3-hBAFF-Luc plasmid and stimulated with TNF-*α* for appropriate time. Luciferase activity of hBAFF promoter was measured by using luminometer. (**i**) MH7A cells were stimulated with TNF-*α* for appropriate time. Cell lysates were prepared and western blotting was used to detect hBAFF (top). Data were the representative of four experiments. Each protein band was quantified by using ImageJ 1.34 (bottom). Data in the bar graph represent the means±S.E.M. *P<0.05; **P<0.01; significant difference as compared with TNF-*α*-untreated control (**b**–**d** bottom, **e** bottom, **g**–**i** bottom)

As HIF-1*α* is associated with the pathogenesis of RA^8, 9^ and BAFF controls RA angiogenesis,^31^ we investigated whether BAFF expression is regulated by HIF-1*α* in FLS. We examined hBAFF and HIF-1*α* expression levels under normoxic conditions, and MH7A cells were treated with various concentrations of TNF-*α* for different times (Figure 2). When MH7A cells were treated with various concentrations of TNF-*α* for 6 h, hBAFF, VEGF, and HIF-1*α* transcript levels increased (Figure 2a). A significant increase in hBAFF expression was confirmed by real-time quantitative polymerase chain reaction (qPCR; Figure 2b). The hBAFF promoter, as judged by a luciferase activity assay, was also significantly and dose-dependently enhanced after a 6 h stimulation with TNF-*α* (Figure 2c), which was confirmed by measuring the hBAFF protein level in MH7A cells (Figure 2d). HIF-1*α* protein levels under normoxic conditions also increased in response to TNF-*α* treatment (Figure 2e). In addition, when MH7A cells were treated with TNF-*α* for different durations, hBAFF, VEGF, and HIF-1*α* transcript levels increased (Figure 2f). A significant increase in hBAFF expression was confirmed by real-time qPCR (Figure 2g). Transcriptional activity of the hBAFF promoter was enhanced significantly and time-dependently after stimulation with TNF-*α* (Figure 2h), which was confirmed by measuring the hBAFF protein level in MH7A cells (Figure 2i). These results suggest that HIF-1*α* may directly or indirectly regulate hBAFF expression.

### TNF-*α*-treated hBAFF expression is dependent on HIF-1*α* expression

**Figure 3:**
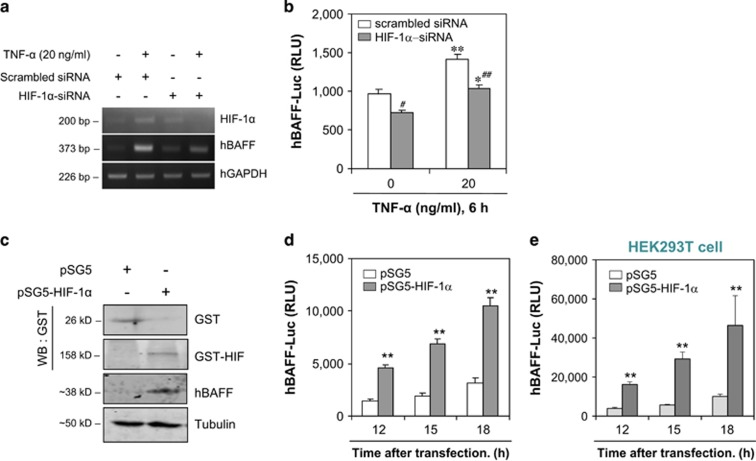
hBAFF transcriptional activity was regulated by HIF-1*α*. (**a**) and (**b**) MH7A cells were transfected with BAFF-siRNA or co-transfected with pGL3-hBAFF-Luc plasmid and HIF-1*α*-siRNA. Then, the cells were stimulated with TNF-*α* and RNA was isolated with TRIzol^TM^. hBAFF transcripts were measured by RT-PCR (**a**). Luciferase activity of hBAFF promoter was measured by using luminometer (**b**). (**c**–**e**) pGL3-hBAFF-Luc and pSG5-HIF-1*α* were co-transfected into MH7A (**c** and **d**) or HEK293T cells (**e**). Cell lysates were prepared and western blotting was used to detect hBAFF, GST, and GST-HIF-1*α* (**c**). Luciferase activity of hBAFF promoter was measured by using luminometer (**d** and **e**). Data were the representative of four experiments. Data in the bar graph represent the means±S.E.M. *P<0.05; **P<0.01, significant difference as compared with pSG5 plasmid-transfected control (**b**–**e**) at each time point (**d** and **e**). ^#^*P*<0.05; ^##^*P*<0.01, significant difference as compared with TNF-α-treated and scambled siRNA-treated control (**b**)

We examined the association between HIF-1*α* and hBAFF expression. When MH7A cells were transfected with HIF-1*α*-siRNA, hBAFF expression was inhibited according to reverse transcriptase polymerase chain reaction (RT-PCR; Figure 3a). When pGL3-hBAFF-Luc plasmids were co-transfected with HIF-1*α*-siRNA into MH7A cells, hBAFF promoter activity was significantly inhibited after stimulation with TNF-*α* for 6 h (Figure 3b). We observed no changes in the toxicity of HIF-1*α*-siRNA-transfected control cells, as judged by a trypan blue exclusion assay (data not shown). No differences in total cell number were detected without TNF-*α* treatment before or after transfection with HIF-1*α*-siRNA (data not shown). These data demonstrate that BAFF expression could be associated with HIF-1*α* in TNF-*α*-stimulated synovial cells.

To confirm whether hBAFF expression is regulated by HIF-1*α*, changes in hBAFF promoter activity were measured in HIF-1*α*-transfected MH7A cells. When pSG5-HIF-1*α* was overexpressed in MH7A cells transfected with the pGL3-hBAFF-Luc plasmid, glutathion-S-transferase (GST) protein from pSG plasmids was detected and BAFF expression was enhanced in pSG5-HIF-1*α*-overexpressing cells (Figure 3c). We observed no changes in toxicity in pSG5-HIF-1*α*-transfected control cells, as judged by the trypan blue exclusion assay (data not shown). No differences were detected in total cell number without TNF-*α* treatment before or after transfection with pSG5-HIF-1*α* (data not shown). hBAFF promoter activity in MH7A cells increased significantly by the overexpression of HIF-1*α* in MH7A cells (Figure 3d), and a significant effect of HIF-1*α* on hBAFF promoter was confirmed in HEK293T cells (Figure 3e).

**Figure 4:**
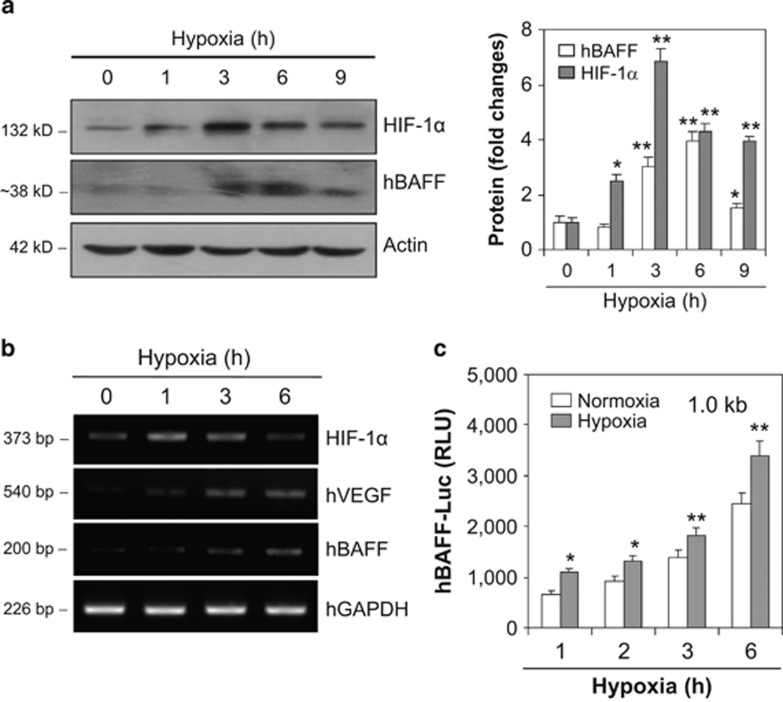
hBAFF expression was increased under hypoxic condition. (**a** and **b**) MH7A cells were incubated for various times under hypoxic condition. RNA was isolated with TRIzol^TM^. hBAFF transcripts were measured by RT-PCR (**a**, left). Each protein band was quantified by using ImageJ 1.34 (**a**, right). Cell lysates were prepared and western blotting was used to detect hBAFF, hVEGF and HIF-1*α* (**b**). (**c**) MH7A cells were transfected with pGL3-hBAFF-Luc plasmids and incubated under hypoxic conditions. Luciferase activity of hBAFF promoter was measured by using luminometer. Data were the representative of four experiments. Data in the bar graph represent the means±S.E.M. *P<0.05; **P<0.01, significant difference as compared with control under normoxia at each time point (**a**, right and **c**)

To re-confirm the effect of HIF-1*α* on hBAFF expression, the MH7A cells were incubated under hypoxic conditions for various times. As shown in Figure 4a, hBAFF and HIF-1*α* protein levels increased, as well as hBAFF, hVEGF, and HIF-1*α* transcript levels (Figure 4b). A significant increase in hBAFF expression was confirmed by an increase in hBAFF promoter activity as judged by luciferase activity assay (Figure 4c). These results suggest that hBAFF production could be controlled by HIF-1*α* expression in synovial cells.

### hBAFF expression is enhanced by HIF-1*α* binding to the MH7A cell hBAFF promoter

**Figure 5:**
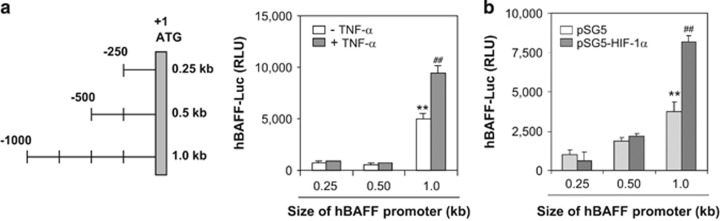
A 1.0 kb hBAFF promoter activity was higher than 0.25 or 0.5 kb in MH7A cells. (**a**) The 0.25 and 0.5 kb pGL3-hBAFF-Luc plasmids were prepared by the deletion of 750 and 500 bp from the 5′-end of 1.0 kb hBAFF promoter, respectively. (**b**) The MH7A cells were transfected with 0.25, 0.5, or 1.0 kb pGL3-hBAFF-Luc plasmids and incubated in the presence or absence of TNF-*α* for 6 h. hBAFF promoter activity was measured as luciferase activity using luminometer. (**c**) pGL3-hBAFF-Luc and pSG5-HIF-1*α* plasmids were co-transfected into MH7A. Luciferase activity of hBAFF promoter was measured by using luminometer. Data were the representative of four experiments. Data in the bar graph represent the means±S.E.M. **P<0.01, significant difference as compared with 0.25 or 0.5 kb hBAFF-Luc-transfected group. ^##^*P*<0.01, significant difference as compared with TNF-*α*-untreated control for each size of hBAFF promoter (**b** and **c**)

Given that hBAFF promoter construct ranges from 0.25 to 1.0 kb,^32^ we examined where HIF-1*α* affects the hBAFF promoter. As shown in Figure 5a, when MH7A cells were transfected with different sizes of hBAFF promoter plasmids, hBAFF promoter activity increased significantly after treatment with TNF-*α*. hBAFF promoter activity increased in response to overexpression of HIF-1*α* when each size of hBAFF promoter plasmid was co-transfected with pSG5-HIF-1*α* (Figure 5b). Transcriptional activity of the 1.0 kb hBAFF promoter was higher than that of the 0.25 or 0.5 kb promoters, suggesting that HIF-1*α* binds the hBAFF promoter from −501 to −1000 bp.

**Figure 6:**
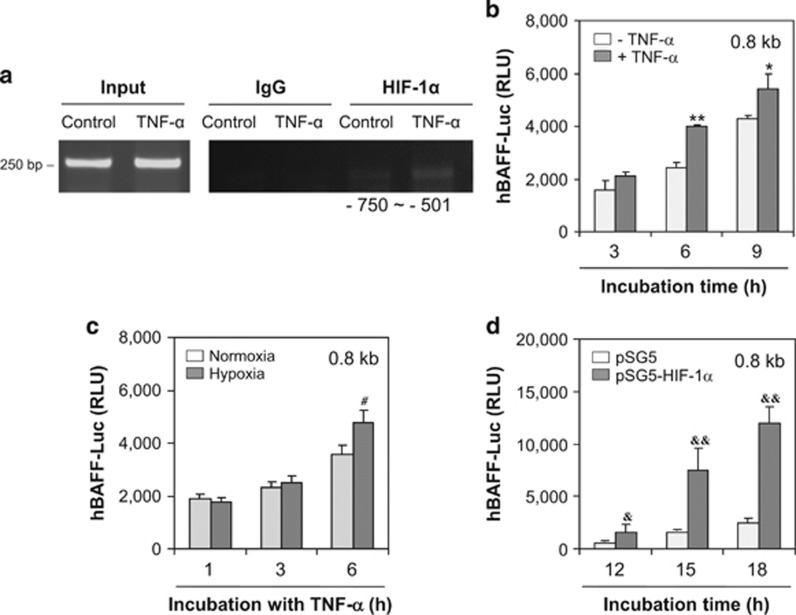
hBAFF expression was enhanced by HIF-1*α* binding in between −750 and −501 bp of hBAFF promoter. (**a**) MH7A cells were stimulated with TNF-*α* and fixed with 1% formaldehyde. Their chromatin extracts were immunoprecipitated with anti-HIF-1*α* antibodies. DNA fragments were subjected to PCR analysis using primer sets spanning the promoter regions. Sequences for primer set were 5′-TTTTCCTTAAAAATATATTC-3′ (forward) and 5′-GTGAAGGTCAGATAAGCT-3′ (reverse). Primer set corresponds to −750 to −501 bp including HIF-1*α* binding site (−693 to −688 bp) on hBAFF promoter. (**b**–**d**) The 0.8 kb pGL3-hBAFF-Luc plasmids were prepared by the deletion of 200 bp from 5′-end of 1.0 kb hBAFF promoter. The MH7A cells were transfected with 0.8 kb pGL3-hBAFF-Luc plasmid (**b** and **c**). Then, the cells were incubated in the presence or absence of TNF-*α* for 6 h (**b**), or under hypoxic condition (**c**). The 0.8 kb pGL3-hBAFF-Luc and pSG5-HIF-1*α* plasmids were co-transfected into MH7A (**d**). Luciferase activity of hBAFF promoter was measured by using luminometer. Data were the representative of four experiments. Data in the bar graph represent the means±S.E.M. *P<0.05; **P<0.01, significant difference as compared with TNF-*α*-untreated control at each time point (**b**). ^#^*P*<0.05, significant difference as compared with control under normoxia condition at each time point (**c**). ^&^*P*<0.05; ^&&^*P*<0.01, significant difference as compared with pSG5 plasmid-transfected control at each time point (**d**)

To examine HIF-1*α* binding to 0.25 kb fragments of the 1.0 kb hBAFF promoter, we performed a chromatin immunoprecipitation (ChIP) assay. As shown in Figure 6a, HIF-1*α* was associated from −750 to −501 bp. However, no association of HIF-1*α* was found from −1000 to −751 bp (data not shown). Then, we deleted the −1000 to −800 bp fragment, beginning at 1.0 kb, to confirm hBAFF promoter activity via HIF-1*α* binding, which resulted in a 0.8 kb hBAFF promoter. When MH7A cells were transfected with the 0.8 kb hBAFF promoter plasmids, hBAFF promoter activity increased significantly after treatment with TNF-*α* (Figure 6b). Activity of the 0.8 kb hBAFF promoter increased significantly after incubation under hypoxic conditions (Figure 6c) or by co-transfection with pSG5-HIF-1*α* (Figure 6d), suggesting that HIF-1*α* binds with the hBAFF promoter from −501 to −750 bp.

**Figure 7:**
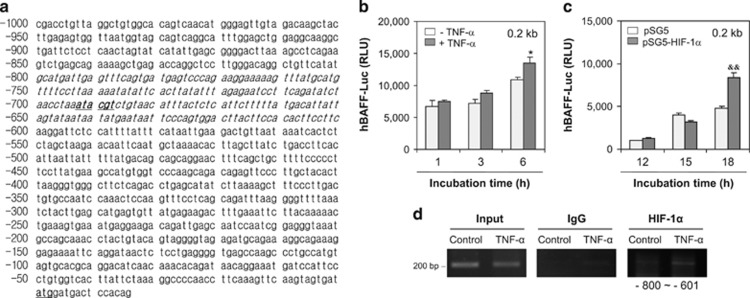
A 0.2 kb hBAFF promoter (−800 to −601 bp) activity was enhanced by TNF-*α* treatment and HIF-1*α* binding. (**a**) Transcription of hBAFF gene started at the site of bold nucleotide with underline on promoter. Italic bold nucleotide with underline showed HIF-1*α* binding site on hBAFF promoter. The 0.2 kb (italic nucleotides, −800 to −601 bp) were cloned into pGL3 plasmids (0.2 kb pGL3-hBAFF-Luc). (**b** and **c**) MH7A cells were transfected with 0.2 kb pGL3-hBAFF-Luc plasmid. Then, the cells were incubated in the presence or absence of TNF-*α* for 6 h (**b**). MH7A cells were co-transfected with 0.2 kb pGL3-hBAFF-Luc and pSG5-HIF-1*α* plasmids (**c**). Luciferase activity of hBAFF promoter was measured by using luminometer. Data were the representative of four experiments. Data in the bar graph represent the means±S.E.M. *P<0.05, significant difference as compared with TNF-*α*-untreated control at each time point (**b**). ^&&^*P*<0.01, significant difference as compared with pSG5 plasmid-transfected control at each time point (**c**). MH7A cells were stimulated with TNF-*α* and fixed with 1% formaldehyde. Their chromatin extracts were immunoprecipitated with anti-HIF-1*α* antibodies. DNA fragments were subjected to PCR analysis using primer sets spanning the promoter regions. Sequences for primer set were 5′-GCATGATTGAGTTTCAGTGA-3′ (forward) and 5′-GAAHGAAGTGTGGAAGTAAG-3′ (reverse). Primer set corresponds to −800 to −601 bp including HIF-1*α* binding site (−693 to −688 bp) on hBAFF promoter. Data were the representative of four experiments (**d**)

We analyzed a TRANSFAC database to examine HIF-1*α* binding to the hBAFF promoter. The results showed that HIF-1*α* bound from −693 to −688 bp on the hBAFF promoter (Figure 7a). Then, we deleted −801 to −1000 bp and −600 to −1 bp, beginning at 1.0 kb, which resulted in a 0.2 kb hBAFF promoter. When MH7A cells were transfected with 0.2 kb hBAFF promoter plasmids, hBAFF promoter activity increased significantly after treatment with TNF-*α* (Figure 7b). Activity of the 0.2 kb hBAFF promoter increased significantly after co-transfection with pSG5-HIF-1*α* (Figure 7c). The ChIP assay showed that HIF-1*α* bound from −800 to −601 bp of the hBAFF promoter in MH7A cells treated with TNF-*α* (Figure 7d). These results suggest that TNF-*α*-mediated BAFF expression was regulated by HIF-1*α* binding to the hBAFF promoter in synovial cells.

### hBAFF expression decreases by inhibiting TNF-*α*-induced ERK with PD98059

**Figure 8:**
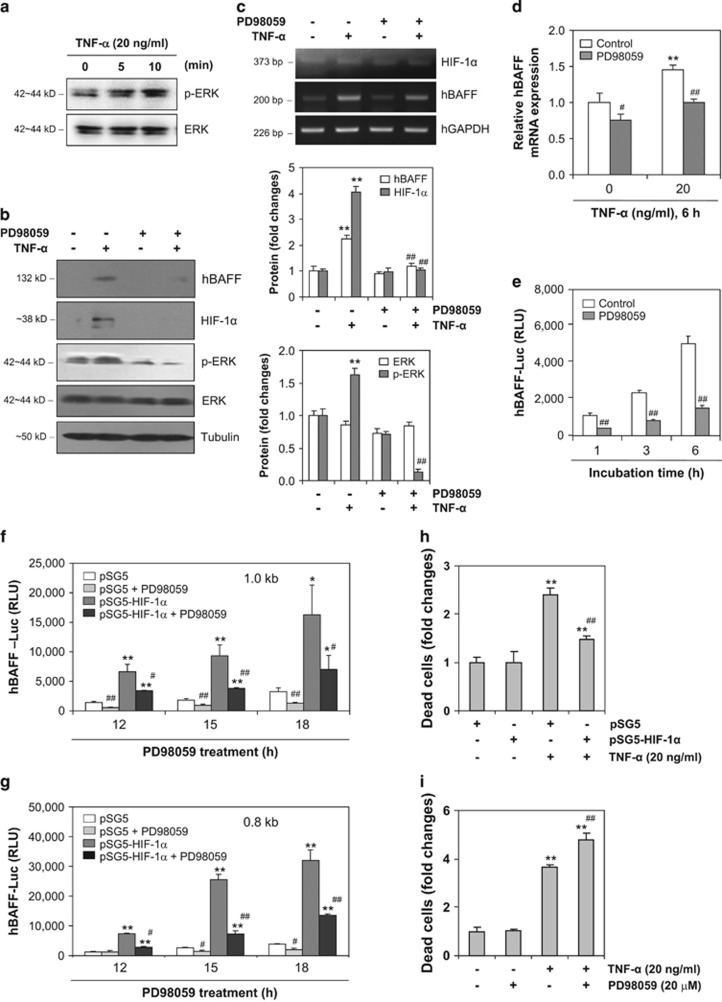
hBAFF expression was dependent on TNF-*α*-induced ERK activation. (**a**–**d**) MH7A cells were stimulated with TNF-*α* in the absence (**a**) or presence (**b**–**d**) of PD98059, ERK inhibitor. Cell lysates were prepared and western blotting was performed to detect p-ERK, ERK (**a** and **b**), hBAFF, or HIF-1*α* (**b**, left). Each protein band was quantified by using ImageJ 1.34 (**b**, right). RNA was isolated with TRIzol^TM^. hBAFF and HIF-1*α* transcripts were measured by RT-PCR (**c**) and real-time qPCR normalized to GAPDH expression (**d**). (**e**–**g**) MH7A cells were transfected with pGL3-hBAFF-Luc plasmid and stimulated with TNF-*α* in the presence or absence of PD98059 (**e**). MH7A cells were co-transfected with 1.0 kb (**f**) or 0.8 kb (**g**) pGL3-hBAFF-Luc and pSG5-HIF-1*α* plasmids. Then, cells were incubated with TNF-*α* in the presence or absence of PD98059 (**f** and **g**). Luciferase activity of hBAFF promoter was measured by using luminometer. (**h** and **i**) MH7A cells were transfected with pSG5 or pSG5-HIF-1*α* (**h**) or pretreated with PD98059,ERK inhibitor (**i**). Then, MH7A cells were stimulated with TNF-*α* for 72 h. Dead cells were estimated with trypan blue exclusion assay. Data were the representative of four experiments. Data in the bar graph represent the means±S.E.M. *P<0.05; **P<0.01, significant difference as compared with TNF-α-untreated control (**b**, right, **d**, **h** and **i**) or pSG5 plasmid-transfected control (**h**) at each time point (**f** and **g**). ^#^*P*<0.05; ^##^*P*<0.01, significant difference as compared with TNF-*α*-treated and PD98059-untreated control (**b**, right, **d**, and **i**), PD98059-untreated control (**d**) at each time point (**e**) with pSG5 or pSG5-HIF-1α plasmid-transfected control (**f** and **g**), or TNF-*α*-treated and pSG5-plasmid-transfected control (**h**)

We assessed ERK phosphorylation to determine whether signaling molecules control hBAFF expression. When the MH7A cells were treated with TNF-*α*, ERK phosphorylation increased significantly in TNF-*α*-treated cells (Figure 8a). When the MH7A cells were treated with TNF-*α* in the presence or absence of the ERK inhibitor PD98059 (Figure 8b, left and right bottom), hBAFF and HIF-1*α* protein levels decreased in response to PD98059 (Figure 8b, left and right top). In addition, hBAFF and HIF-1*α* transcript levels were inhibited by PD98059 treatment (Figure 8c). PD98059 significantly inhibited hBAFF expression according to real-time qPCR (Figure 8d). Transcriptional activity of the 1.0 kb hBAFF promoter was enhanced by TNF-*α* stimulation, but was also inhibited significantly by treatment with PD98059 (Figure 8e); this suggests that hBAFF expression may be regulated by HIF-1*α* via activation of ERK. The results were confirmed by co-transfection of the 1.0 or 0.8 kb hBAFF promoter plasmid with the pSG5-HIF-1*α* plasmid. hBAFF promoter activity, which was increased by overexpression of HIF-1*α*, was significantly inhibited by treatment with PD98059 (Figures 8f and g). These data suggest that hBAFF expression may be associated with HIF-1*α* binding to the hBAFF promoter through an ERK-mediated increase in HIF-1*α* level.

### Survival of synovial cells was recovered by PD98059 treatment or HIF-1*α* overexpression

We examined the association between HIF-1*α* and death of synovial cells. When MH7A cells were transfected with the pSG5-HIF-1*α* plasmid and treated with TNF-*α*, death of synovial cell decreased compared with that in the pSG5-transfected TNF-*α*-treated control (Figure 8h). We also examined whether ERK controlled the death of synovial cells. When MH7A cells were treated with TNF-*α* in the presence or absence of PD98059, cell death was increased by pre-treatment with PD98059 compared with that in the TNF-*α*-induced cell death control (Figure 8i). These results suggest that the survival of synovial cells may be regulated by TNF-*α*-induced hBAFF expression via activation of ERK and the expression and binding of HIF-1*α* to the hBAFF promoter.

## Discussion

RA is characterized by synovial hyperplasia and destruction of cartilage and bone.^1^ Various immune cells are recruited and accumulate in joint tissues.^3^ Synovial macrophages and FLS in patients with RA secrete abundant pro- and anti-inflammatory cytokines, including TNF-*α*.^3, 16, 17, 22^ Then, RA deteriorates in response to increased production of TNF-*α* and other cytokines.^18, 19^ The abnormal increase in the number of synoviocytes decreases oxygen tension,^4, 5^ stabilizes HIF-1*α*, and induces VEGF expression.^9, 13, 14^ HIF-1*α* is also stabilized by TNF-*α* under normoxic conditions.^20^ In addition, TNF-*α*-stimulated FLS express BAFF, a well-known B-cell proliferation and survival factor,^23^ which may contribute to RA angiogenesis by regulating VEGF expression.^31^ Autoimmunity can deteriorate due to an increase in B-cell survival and excess B-cell responses, caused by the expression of BAFF, which increases in response to TNF-*α* stimulation.^27, 29, 30^ Although HIF-1*α* and BAFF are highly expressed in the joints of patients with RA, little has been reported about the relationship between these two factors. Here, we report the effect of TNF-*α*-induced BAFF expression on the survival of synovial cells, as well as the mechanism of action of BAFF expression in a HIF-1*α*-dependent manner in FLS.

BAFF was originally known as a B-cell proliferation and survival factor.^23^ BAFF is not only produced by myeloid cells,^24, 33^ but also by non-lymphoid cell types.^27, 31, 34, 35, 36, 37^ However, with the exception of B cells, little is known about the role of BAFF on other types of cell, including myeloid cells and non-lymphoid cells. We observed a significant increase in hBAFF expression, in the intracellular compartment and on the cell surface, when synovial cells were treated with TNF-*α* (Figures 1a–c and Figure 2). As synovial hyperplasia is a characteristic of RA, our data show that TNF-*α*-induced death of synovial cells was reduced by inhibiting apoptosis with Z-VAD treatment, which enhanced hBAFF expression (Figures 1d–f). The role of hBAFF in the survival of synovial cells was confirmed by BAFF-siRNA (Figures 1g and h), suggesting that hBAFF expression might be associated with regulating the survival of synovial cells in patients with RA.

Additional proinflammatory cytokines, such as TNF-*α*, are produced by hypoxia-exposed macrophages.^15^ We observed the changes of BAFF transcripts at 1, 3, 6, 9 h-incubated synocytes with TNF-*α* (Figure 2f) and we choose only the time point to show the highest hBAFF expression level at 6 h-incubation with TNF-*α*. Even though protein generally changes following transcript changes, our data showed that the transcriptional increase looks like at the same time to protein increase. Then, we just would like to figure out the regulatory mechanism of hBAFF on synovial cell death via HIF-1*α*.

HIF-1*α* accumulates even under normoxic conditions in an inflammatory condition.^11^ The high expression level of HIF-1*α* detected in synoviocytes may have a role in the pathogenesis of RA.^8, 9^ Therefore, TNF-*α*-induced BAFF expression could be regulated by the association between HIF-1*α* and the BAFF promoter. Our data show that the expression of HIF-1*α* and VEGF increased under various experimental conditions used to treat synoviocytes with TNF-*α* (Figure 2). Although transfection with HIF-1*α*-siRNA inhibited hBAFF expression, overexpression of pSG5-HIF-1*α* increased hBAFF expression (Figure 3). Our results also show that the hBAFF promoter was activated by the binding of HIF-1*α*. Increased hBAFF expression was confirmed under hypoxic conditions (Figure 4), suggesting that hBAFF could be a novel target gene for HIF-1*α*-mediated transcription.

A previous report showed that BAFF expression is associated with inhibited lung function and hypoxia in patients with chronic obstructive pulmonary disease,^38^ suggesting a link between hypoxia and the induction of BAFF in FLS. As HIF-1*α* is a well-known transcription factor,^6, 7^ we investigated the role of HIF-1*α* in the regulation of BAFF promoter activity and BAFF expression by overexpressing HIF-1*α*. Our data show that HIF-1*α* may be associated with the −750 to −501 bp region of the hBAFF promoter (Figures 5 and 6). HIF-1*α* binding to the hBAFF promoter was demonstrated by detecting the −800 to −601 bp region in MH7A cells treated with TNF-*α* (Figure 7). These results suggest that HIF-1*α* may regulate hBAFF expression directly or indirectly, and may also be associated with the pathogenesis of RA. Thus, hBAFF expression could be regulated by the binding of HIF-1*α* to a specific site on the BAFF promoter.

BAFF expression is also regulated by various signaling pathways, including the activation of nuclear factor-kappa B by lipopolysaccharide (LPS)-induced production of reactive oxygen species (ROS),^39^ the CREB-binding protein/p300,^40, 41, 42^ JAK/STAT,^35^ LPS-induced protein kinase A-mediated CREB, the Epac1-mediated Rap1,^43, 44^ and ROS-dependent protein kinase C (PKC)/c-Fos pathways. Both the ERK and phosphoinositide 3-kinase pathways are involved in IL-1*β* and TNF-*α*-induced HIF-1*α* expression in FLS.^45^ Our data show that hBAFF expression could be associated with HIF-1*α* binding to the hBAFF promoter through the ERK-mediated increase in HIF-1*α* level (Figure 8). The ERK/HIF-1*α* pathway in our study could be an additional signaling pathway to control hBAFF expression.

Taken together, although it has not been cleared of all possible signaling molecules for TNF-*α*-induced BAFF expression except ERK, our data suggest that the survival of synovial cells could be regulated by BAFF expression through the association between HIF-1*α* and the BAFF promoter. In addition, it appears that TNF-*α*-induced BAFF expression in FLS may protect synovial cells by themselves and mediate a crosstalk to protect B cells from apoptosis in the inflammatory microenvironment of RA. These results may for the first time provide a novel role for BAFF in cells other than B cells. Data also suggest that HIF-1*α* could be a novel target for treating RA.

## Materials and methods

### Reagents

Recombinant human TNF-*α* was purchased from R&D System Inc. (Minneapolis, MN, USA). Anti-BAFF antibody was obtained from Sigma-Aldrich (St. Louis, MO, USA), anti-HIF-1*α* and ERK antibodies were from Santa Cruz Biotechnology, Inc. (Santa Cruz, CA, USA), and phospho-p44/42 (ERK1/2) was from Cell Signaling Technology (Beverly, MA, USA). Anti-human CD257 (BAFF, BLyS) biotin antibody was come from eBioscience (San Diego, CA, USA). Small interference RNA for HIF-1*α* was synthesized by Bioneer Inc. (Daejeon, Korea). PD98059, specific inhibitor of ERK, was purchased from Sigma-Aldrich and dissolved in dimethyl sulfoxide (DMSO). Z-VAD was purchased from Calbiochem (Santa Diego, CA, USA). Reporter lysis buffer and luciferase substrate were obtained from Promega (Madison, WI, USA).

### Cloning hBAFF gene promoter

Human BAFF(hBAFF) promoter (1 kb) upstream (AF116456.1) from the starting codon (ATG) was searched from NCBI database (AL157762.13). Primers for various sizes of hBAFF promoter were designed from the sequence; forward primer including SacI site (5′-GAGCTCCGACCTGTTAGGCTGT-3′ for 1 kb; 5′-GGAGCTCTTTTCCTTAAAAATATATTC-3′ for 0.75 kb; 5′-GAGCTCATTAATTATTTTTATGACAGC-3′ for 0.5 kb and 5′-GAGCTCTGAAAGTGAAATGAGGAAGAC-3′ for 0.25 kb) and reverse primer including BglII site (5′-GGAGATCTATCACTACTTGAACTTTGAAGG-3′ for all size of promoters). hBAFF promoters (0.2 or 0.8 kb) including HIF-1*α* binding site were respectively cloned from −800 bp to −601 or −1 bp in 1 kb sequences with forward primer including SacI site (5′-GAGCTCGCATGATTGAGTTTCAGTGA-3′ for both 0.2 and 0.8 kb) and reverse primers including BglII site (5′-GGAGATCTGAAGGAAGTGTGGAAGTAAG-3′ for 0.2 kb or 5′-GGAGATCTATCACTACTTGAACTTTGAAGG-3′ for 0.8 kb). Each size of upstream sequence was amplified from human peripheral blood mononuclear cell's chromosomal DNA by PCR. Each promoter was cloned into the site between SacI and BglII of pGL3 plasmid that contains firefly luciferase (pGL3-hBAFF-Luc). Each product was sequenced and matched to NCBI database.

### Collection of human RA-FLS

Informed consent was obtained from all patients, and the experimental protocol was approved by the Konkuk University Medical Center Institutional Review Board. RA patients fulfilled the criteria of the American College of Rheumatology (formerly, the American Rheumatism Association).^46^ RA-FLS were isolated from the synovial tissues according to a protocol as follows. Briefly, synovial tissues were washed thoroughly with RPMI 1640 (Gibco BRL, Gaithersburg, MD, USA), minced into 1 mm^3^, and digested for 90 min at 37 °C in RPMI 1640 containing 1 mg/ml collagenase (Gibco BRL). The digested tissue was filtered with a 70 *μ*m cell strainer (Becton Dickinson, Franklin Lakes, NJ, USA), and centrifuged at 250 × *g* for 10 min. The cell pellet was resuspended in RPMI 1640, washed three times by centrifugation, and suspended in *α*-minimum essential medium (*α*-MEM; Irvine Scientific, Santa Ana, CA, USA) containing 10% fetal bovine serum (FBS; Gibco BRL). The cells were then subcultured for three to six passages before use.

### Cell cultures

MH7A synovial cells isolated from intra-articular soft tissues of the knee joints of RA patients were obtained from the Riken cell bank (Ibaraki, Japan) through Dr. Ho-Geun Yoon of Yonsei University (Seoul, Korea). Briefly, MH7A is a cell line established by transfection with the SV40 T antigen.^47^ MH7A cells were cultured in RPMI 1640 (Gibco BRL, USA) supplemented with 10% heat-inactivated FBS, penicillin (final concentration, 100 U/ml), streptomycin (P/S, final concentration, 0.1 mg/ml) and l-glutamine at 37 °C in an atmosphere of 5% CO_2_ in air.

### Measurement of hBAFF promoter activity

The MH7A cells were transfected with pGL3-hBAFF-Luc and pcDNA-lacZ for monitoring transfection efficiency by *β*-galactosidase assay. Luciferase activity was determined by incubating cell extracts with luciferase substrate (Promega). Luminescence was measured using luminometer (Berthold Technologies, Oak Ridge, TN, USA). Luciferase units of experimental vector were normalized to the control vector in each sample.^31, 32, 48^

### Hypoxia treatment

For incubation under hypoxic conditions, cells were placed in an atmosphere of 1% O_2_, 5% CO_2_, 10% H_2_, and 84% N_2_ with intermittent flushing with nitrogen, sealed, and then maintained in a humidified incubator at 37 °C in a hypoxia chamber (Forma Anaerobic System, Thermo Electron Corporation). Hypoxia-treated cells were collected in the hypoxia chamber to prevent the rapid degradation of hypoxia-responsive molecules.

### siRNA transfection

siRNA for HIF-1*α* (GenBank accession number NM_001530.2 and NM_181054.1) and BAFF was purchased from Bioneer (Daejeon, Korea). When cells were 70–80% confluent in a 12-well plate, the medium was changed with serum-free RPMI 1640. Transfection was performed with lipofectamine 2000 transfection reagent according to the manufacturer's protocol (Invitrogen, Carlsbad, CA, USA). Briefly, 20 pmol of each siRNA or 1 *μ*l of lipofectamine 2000 transfection reagent diluted in 50 *μ*l serum-free RPMI 1640 was mixed after the incubation for 5 min. The mixture was incubated for the additional 20 min and carefully added to the cells. Then, the cells were incubated for 4 h and the medium was changed again with a complete RPMI 1640 containing 10% FBS, P/S, and l-glutamine. The cells were collected at 48 h for RNA isolation and protein extraction.

### Immunostaining

The levels of hBAFF in MH7A cells was visualized by immunostaining. Briefly, cells were treated with TNF-*α*, fixed with 2% paraformaldehyde, permeabilized with 0.2% Triton X-100 in PBS and incubated with following antibodies. Biotinylated anti-CD257 (BAFF, BLyS) antibodies (0.3 *μ*g/100 *μ*l) and PE-conjugated streptavidin (0.15 *μ*g/100 *μ*l) were used for immunostaining with the dilution in PBS containing 0.01% Triton X-100 and 1% BSA. The cells were washed, mounted and observed with x400 magnification under fluorescence microscope.

### FACS analysis

hBAFF protein expression was examined by flow cytometry analysis. MH7A cells were treated with TNF-*α*, collected and washed twice with 1 × HBSS containing 1% bovine calf serum (BCS). For the detection of hBAFF on cell surface by flow cytometer, the cells were incubated with bionylated anti-CD257 antibodies for 1 h. After two times wash with 1 × HBSS containing 1% BCS, cells were incubated with streptavidin-PE (BD Pharmingen, San Jose, CA, USA) for 30 min. For the detection of total amount of hBAFF by flow cytometer, cells were incubated with fixation/permeabilization solution (eBioscience), washed with PBS. Then, cells were incubated again with biotinylated anti-CD257 antibodies (0.25 *μ*g/100 *μ*l) and streptavidin-PE (0.1 *μ*g/100 *μ*l) diluted in 1 × permeabilization buffer (eBioscience) or 1 X HBSS containing 1% BCS. Fluorescence intensity of 10,000 cells was measured by FACSCalibur flow cytometer (BD Bioscience, San Jose, CA, USA). Data were analyzed using WinMDI 2.8 software (http://facs.scripps.edu/software.html).

### Chromatin immunoprecipitation assay

Chromatin immunoprecipitation (ChIP) assays were performed as described previously.^49, 50^ The cells were crosslinked with final concentration 1% formaldehyde for 10 min at room temperature. Then 125 mM glycine was added to quench unreacted formaldehyde. The cells were gathered and sonicated to make DNA fragments with a size range of 200 to 1000 bp. The cell extracts were immunoprecipitated using 2 *μ*g anti-HIF-1*α* or rabbit IgG control (Abcam, Cambridge, UK) for each sample suspended in 450 *μ*l ChIP dilution buffer (0.01% SDS, 1.1% Triton X-100, 1.2 mM EDTA, 16.7 mM Tris-HCl, pH 8.1, 167 mM NaCl) purchased from Cell Signaling Technology (Cat #20–153, Danvers, MA, USA). For all ChIP experiments, PCR analysis were performed by using multiple sets of primers spanning the transcription factor binding site on hBAFF gene promoter.

### Reverse transcriptase polymerase chain reaction

Total RNA was extracted from MH7A cells using TRIzol^TM^ reagent (Invitrogen). cDNA was synthesized from 1 *μ*g of total RNA using oligo-dT_18_ primers and reverse transcriptase in a total volume of 21 *μ*l (Bioneer, Daejeon, Korea). For standard PCR, 1 *μ*l of the first-strand cDNA product was then used and 10 pmol of specific primers were used as a template for PCR amplification with Taq DNA polymerase (Cosmo Genetech, Seoul, Korea). PCR amplification was performed using primers specific for hBAFF (forward; 5′-AATTCAGAGGAAGAAGGTCC-3′, reverse; 5′-ATGTGACATCTCCATCCAGT-3′) with 36 cycles (95 °C for 40 s, 57 °C for 30 s and 72 °C for 60 s), hHIF-1*α* (forward: 5′-CTCAAAGTCGGACAGCCTCA-3′, reverse: 5′-GATTGCCCCAGCAGTCTACA-3′), hVEGF (forward: 5′-TGACAGGGAAGAGGAGGAGA-3′, reverse: 5′-TGGTTTCAATGGTGTGAGGA-3′) with 30 cycles (95 °C for 40 s, 55 °C for 30 s and 72 °C for 30 s), and hGAPDH (forward; 5′-ACAAACCCGATATGGCTGAGATCGAGAA-3′, reverse; 5′-CTTGCTTCTCCTGTTCAATC-3′) with 28 cycles (95 °C for 30 s, 55 °C for 30 s and 75 °C for 35 s). PCR products were detected by agarose gel electrophoresis.

### Real-time quantitative PCR analysis

To perform real-time quantitative PCR (qPCR), total cellular RNA (1 *μ*g) was reverse transcribed into cDNA as described in RT-PCR. Real-time qPCR was performed using the CFX96 Touch Real-Time PCR Detection System (Bio-Rad Laboratories, Hercules, CA, USA). The RT reaction product (10 ng) was amplified with Thunderbird SYBR qPCR mix (TOYOBO Co. Ltd., Osaka, Japan) using primers specific for target genes, hBAFF primers (forward; 5′-ACAGAAAGGGAGCAGTCA-3′ and reverse; 5′-TGGGAGGATGGAAACACA-3′), and hGAPDH primers (forward; 5′-GTATGACAACAGCCTCAAGA-3′, reverse; 5′-AGTCCTTCCACGATACCAAA-3′). The samples were heated to 95 °C for 1 min and amplified for 40 cycles (95 °C for 10 s, 55 °C for 10 s and 72 °C for 30 s) followed by a final extension step of 72 °C for 10 min. GAPDH was used as an internal control. Relative quantification of each mRNA was analyzed by the comparative threshold cycle (CT) method and normalized to GAPDH expression using Bio-Rad CFX Manager Software.

### Western blot analysis

Western blotting was performed using a standard protocol. The cells were lysed in ice-cold lysis buffer containing 0.5% Nonidet P-40 (vol/vol) in 20 mM Tris-HCl (pH 8.3); 150 mM NaCl; protease inhibitors (2 *μ*g/ml) aprotinin, pepstatin, and chymostatin; 1 *μ*g/ml leupeptin and pepstatin; 1 mM phenylmethyl sulfonyl fluoride (PMSF); and 1 mM Na_4_VO_3_. Lysates were incubated for 1 h on ice before centrifugation at 13 000 r.p.m. for 10 min at 4 °C. Proteins in the supernatant were measured using a Bio-Rad protein assay dye reagent and denatured by boiling for 5 min in sodium dodecyl sulfate (SDS) sample buffer. Proteins were separated by 12% SDS-polyacrylamide gel electrophoresis (SDS-PAGE), and transferred to nitrocellulose membranes by electroblotting. Following transfer, equal loading of protein was verified by Ponceau staining. The membranes were blocked with 5% skim milk in Tris-buffered saline with Tween 20 (TBST; 10 mM Tris-HCl, pH 7.6; 150 mM NaCl; 0.5% Tween 20) and incubated with the indicated antibodies. The bound antibodies were visualized with HRP-conjugated secondary antibodies with the use of enhanced chemiluminescence (ECL; Pierce, Rockford, IL, USA). Primary anti-BAFF and HRP-labeled secondary anti-IgG antibodies were diluted 1:1000 and 1:5000, respectively in TBST containing 0.5% Tween 20. Immunoreactive bands were detected using X-ray film.

### Statistical analyses

Experimental differences were examined using ANOVA and Students' *t*-tests, as appropriate. *P*-values <0.05 were considered to indicate significance.
